# Case report: A novel *PPP3CA* truncating mutation within the regulatory domain causes severe developmental and epileptic encephalopathy in a Chinese patient

**DOI:** 10.3389/fneur.2022.889167

**Published:** 2022-09-07

**Authors:** Jieling Li, Jie Cao

**Affiliations:** ^1^Department of Medical General Ward, Ministry of Education Key Laboratory of Child Development and Disorders, National Clinical Research Center for Child Health and Disorders, China International Science and Technology Cooperation Base of Child Development and Critical Disorders, Chongqing, China; ^2^Chongqing Key Laboratory of Pediatrics, Children's Hospital of Chongqing Medical University, Chongqing, China

**Keywords:** *PPP3CA*, DEE91, epilepsy, regulatory domain, case report

## Abstract

**Introduction:**

Developmental and epileptic encephalopathy 91 (DEE91; OMIM#617711) is a severe neurodevelopmental disorder caused by heterozygous *PPP3CA* variants. To the best of our knowledge, only a few DEE91 cases have been reported.

**Results:**

This study reports a boy who experienced recurrent afebrile convulsions and spasms at the age of 2 months. After being given multiple antiepileptic treatments with levetiracetam, adrenocorticotropic hormone (ACTH), prednisone, topiramate, and clonazepam, his seizures were not completely relieved. At the age of 4 months, the patient exhibited delayed neuromotor development and difficulty in feeding; at the age of 6 months, he was diagnosed with developmental regression with recurrent spasms and myoclonic seizures that could respond to vigabatrin. At the age of 1 year and 4 months, the patient showed profound global developmental delay (GDD) with intermittent absence seizures. Whole-exome sequencing (WES) identified a novel loss-of-function variant c.1258_1259insAGTG (p. Val420Glufs^*^32) in *PPP3CA*.

**Conclusion:**

This finding expands the genetic spectrum of the *PPP3CA* gene and reinforces the theory that DEE91-associated truncating variants cluster within a 26-amino acid region in the regulatory domain (RD) of *PPP3CA*.

## Introduction

Developmental and epileptic encephalopathy 91 (DEE91; MIM: #617711) is a severe, early-onset neurodevelopmental disease. Patients with DEE91 tend to be clinically diagnosed with West syndrome (WS) or infantile spasms syndrome, developmental regression, and hypsarrhythmia (1, 2). DEE91 is characterized by a delay in infantile neuromotor development, resulting in severely to a profoundly impaired intellectual development, refractory epilepsy, and developmental regression (3). Unlike the diverse genetic causes of WS (2), DEE91 is a well-defined, *PPP3CA*-associated monogenic disease. Thus, the clinical diagnosis of DEE91 is dependent on genetic tests.

Calcineurin is a serine/threonine protein phosphatase that is dependent on calcium and calmodulin. It is a heterodimeric protein consisting of the catalytic subunit calcineurin A and the tightly bound calcium ion-binding subunit calcineurin B. The primary sequences of subunits and heterodimeric quaternary structures are highly conserved from yeast to mammals (4–6). *PPP3CA*, which is also known as calcineurin A, is a catalytic subunit of calcineurin that mediates Ca2^+^-dependent signal transduction (7, 8). Calcineurin defects are associated with various human disorders (9–12). Notably, the heterozygous loss-of-function (LOF) and gain-of-function variants (GOF) in the *PPP3CA* gene are presumed to cause DEE91 and multiple congenital abnormalities (ACCIID; MIM: #618265), respectively (1). Mizuguchi et al. (1) suggested that variants in the catalytic or metal binding domains led to nonsyndromic epileptic encephalopathy, which is related to LOF, while variants in the AI domain cause ACCIID, which belongs to GOF (1).

In this study, we reported a 1 year and 4 month-old male patient who was clinically diagnosed with EE and had refractory epilepsy. During the patient's treatment, seizure progression, developmental delay, and the onset of developmental regression were observed.

## Case description

We reported a 1 year and 4 month-old male patient who was the fourth pregnancy and the second birth to a normal non-consanguineous couple. His perinatal period was normal. The second pregnancy was aborted due to non-medical reasons, and the third pregnancy was terminated at 5 months of pregnancy because of cardiac dysplasia. The elder sister presents no abnormal conditions at the age of 20 years, and there is no family history of disease.

**Figure 1:**
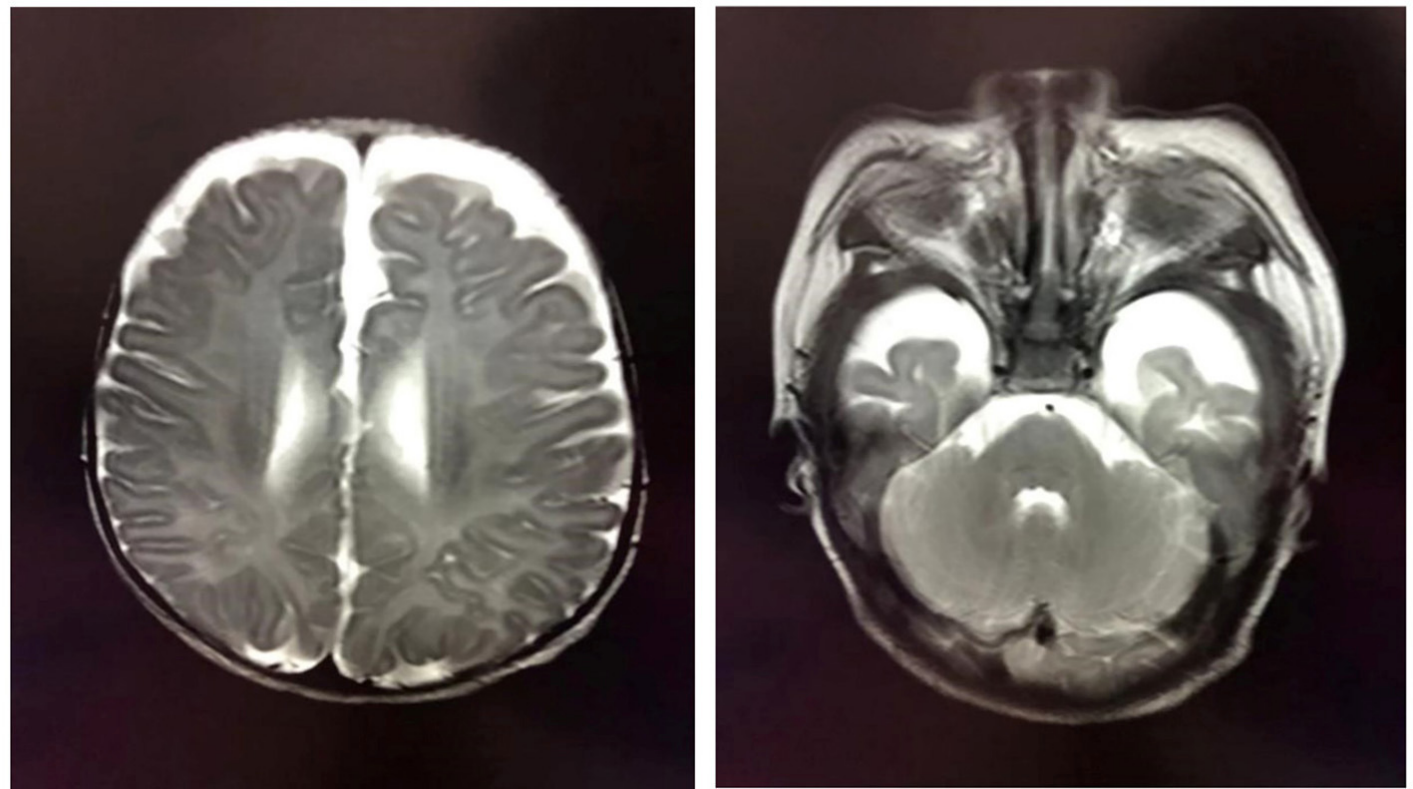
Brain MRI showing the broadened bilateral frontotemporal extracerebral space, which is especially apparent on the left side.

**Figure 2:**
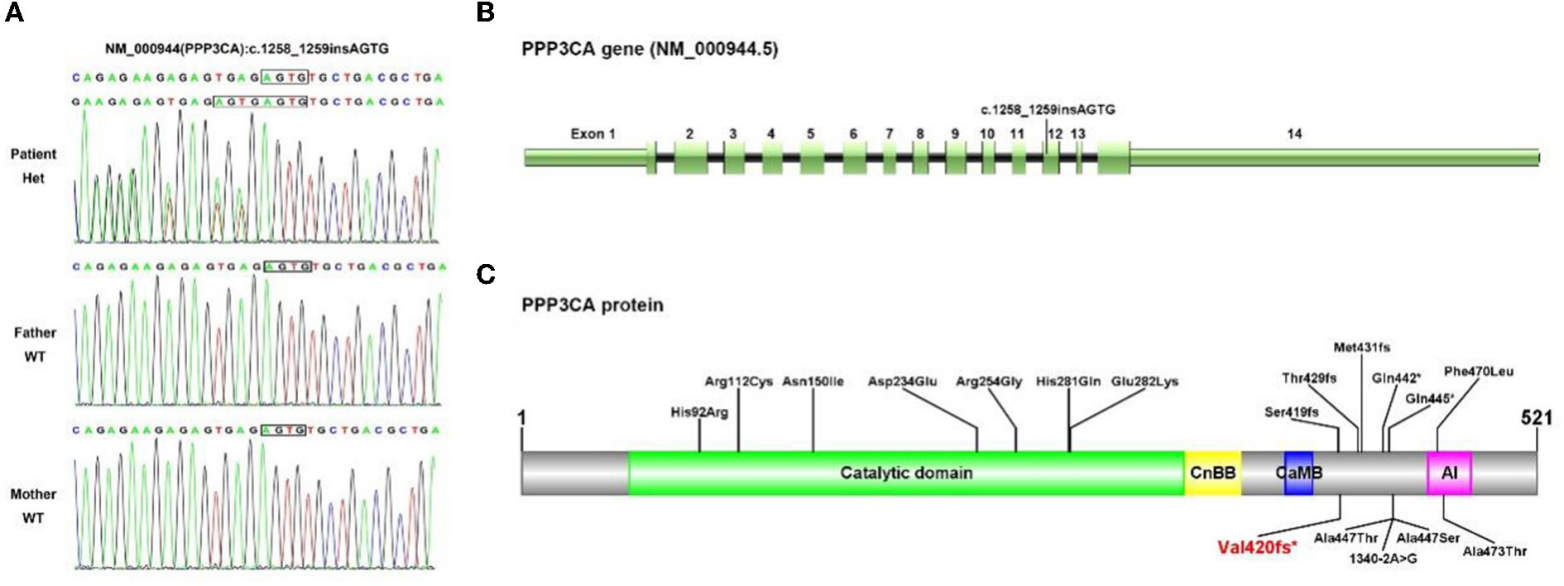
EEG of the patient diagnosed with developmental and epileptic encephalopathy 91 at ages of 2 months **(A)**, 2 and a half months **(B)**, and one year and four months **(C)**.

At the age of 2 months, the patient was admitted to a hospital after experiencing 3 days of recurrent afebrile convulsions. The seizures lasted approximately 10 s every time, occurred five to six times per day, and involved staring eyes, rigid limbs, and cyanosis face and lips, and the patient was unresponsive without incontinence during the attacks. His physical examination and developmental assessment were normal. A brain magnetic resonance imaging (MRI) showed that the bilateral frontotemporal extracerebral space was broadened, especially on the left side (Figure 1). An electroencephalography (EEG) showed multifocal discharges of irregular sharp-slow waves in the frontopolar, lateral frontal, central, and temporal regions during both consciousness and sleep (Figure 2A). The results of laboratory tests were unavailable. The patient was diagnosed with epilepsy and treated with 10 mg/kg/day levetiracetam, divided by q12H, and the dose was added to 20 mg/kg/day at day 3 of the antiepileptic treatment. His convulsions were relieved, the patient was discharged, and 10 mg/kg/day levetiracetam was prescribed as a maintenance dose.

After levetiracetam was administered for ~15 days, the patient developed 10–20 spasms/spasticity in a cluster per day. The features of the spasms included instant onset of involuntary nodding, forward flexion of head and torso, eyes staring upwards, and unresponsiveness. He was then sent for medication for a second time. His physical examination and laboratory tests were normal. An EEG showed modified hypsarrhythmia (Figure 2B). The patient was diagnosed with WS (infantile spasms syndrome) and treated with ACTH at 3 units/kg/day through an i.v. for 12 days, an antiepileptic treatment of 3 mg/kg/day topiramate, divided by q12H, and 0.03 mg/kg/day clonazepam. Levetiracetam was sequentially added at 10 mg/kg/day with divided q12H. His spasms were relieved, the frequency of attacks was reduced to 2–3 times per day, milder involuntary nodding and spasticity were observed, and no facial cyanosis occurred. The patient was discharged with a prescription of prednisone at 1 mg/kg/day, p.o., instead of the i.v. ACTH treatment, and topiramate, clonazepam, and levetiracetam were administered as antiepileptic medication.

At the age of 4 months, the patient was found to have significantly delayed neuromotor development. He was unable to look up or turn over, and he lost the ability to pronounce vowels, accompanied by hypotonia in the limbs. At the age of 6 months, the patient lost a previous developmental milestone, which was manifested as unstable neck erection, and this was accompanied by feeding difficulties, including an unwillingness to eat and refusal to drink milk, as well as recurrent spasms and bilateral tonic-clonic. For the third time, he was treated by adding 45 mg/kg/day vigabatrin divided by q12H instead of levetiracetam, and maintenance doses of topiramate, clonazepam, and prednisone were administered. At the age of 8 months, 60 mg/kg/day vigabatrin, divided by q12H, and 0.1 mg/kg/day clonazepam, divided by q12H, were added to the treatment, and the patient's seizures were significantly relieved but he still experienced hypotension in his limbs.

At the last follow-up examination, the patient was 1 year and 4 months old. The patient weighed 9 kg and had no significant deformity. His motor and language developmental milestones equalled those of normal 1- and 3-month-old infants, respectively, indicating that profound global developmental delay (GDD), including an unstable neck erection and inability to raise his head, turn over, and laugh, occurred with limb hypotension. The patient could only pronounce vowels. Under a treatment with 45 mg/kg/day vigabatrin, divided by q12H, 0.1 mg/kg/day clonazepam, divided by q12H, 6 mg/kg/day topiramate, divided by q12H, and 1 mg/kg/day prednisone, involuntary nodding and forward flexion of head and torso in the patient disappeared; however, he still had mild seizures (with staring eyes for approximately 10 s every time). A follow-up EEG showed modified hypsarrhythmia during sleep (Figure 2C).

## Methods

### Whole-exome sequencing

The whole-exon capture chip used by Trio-WES is the IDT xGen Exome Research Panel version 1.0, which was sequenced by an Illumina NovaSeq 6000 series sequencer (PE150). The sequencing coverage of the target sequence was not <99%. The raw sequencing data were screened, and low-quality reads were filtered through a quality control (QC). Then, the BWA software was used to perform a sequence alignment with the reference human genome. Repeated read operations were excluded, the remaining read operations were statistically analyzed, and the GATK software was used to identify variables. Detected variants were bioinformatically annotated based on public databases such as ClinVar, HGMD, gnomAD, and the 1,000 Genomes Project and were predicted to have pathogenic or deleterious effects based on database data. Variation classification mainly refers to the 2015 ACMG Genetic Variation Classification Standards and Guidelines gene mutation classification system.

## Results

**Figure 3:**
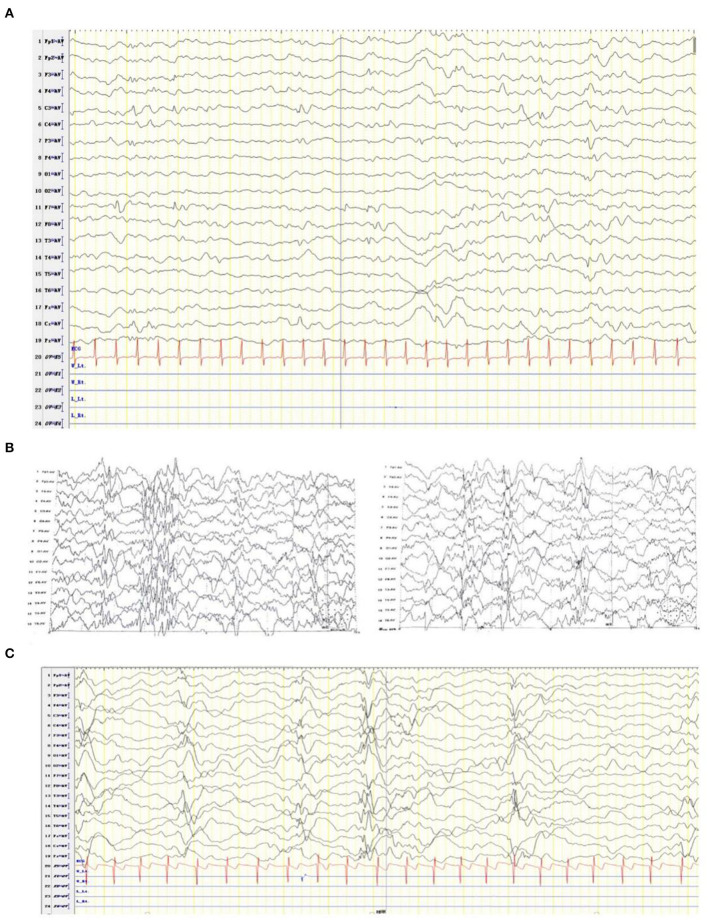
Sanger sequencing **(A)** confirmed the mutation NM_000944.5(PPP3CA):c.1258_125 9 insAGTG on exon 12 **(B)** and the pattern diagram of PPP3CA functional domains **(C)**.

Trio-whole-exome sequencing (WES) was performed after signed consent was obtained from the parents, according to the medical ethics statement. We identified a heterozygous variant (NM_000944.5: c.1258_1259insAGTG, p. Val420Glufs^*^32) of the *PPP3CA* gene exon 12 in the proband. The variant was *de novo*. The result was confirmed by Sanger sequencing (Figure 3A). The variant p. Val420Glufs^*^32 was pathogenic (PVS1 + PS2 + PM2) according to the American College of Medical Genetics and Genomics (ACMG) practical guidelines (16). The variant has not been documented in public variant databases or reported in historical literature. In addition, pathogenic variants in the list of the OMIM genes (https://www.omim.org/phenotypicSeries/PS308350) associated with WS or developmental and epileptic encephalopathies were excluded by WES.

## Discussion and conclusion

We identified a frameshift mutation in *PPP3CA* (p. Val420Glufs^*^32) that caused DEE91, an early-onset WS-like disorder, in a Chinese male pediatric patient. The specific type of seizure, infantile spasms, and developmental regression were significant in our patient, which prompted the initial diagnosis of WS. In detail, infantile spasms, myoclonus, and multifocal hypsarrhythmia were observed in the EEG in the present case, and these observations share the core phenotype of WS (2). However, the onset of infantile spasms in WS cases usually occurs between 4 and 8 months of age (2), while patients with DEE91 have highly variable epilepsy. Of the 6 cases of DEE91 previously reported by Myers et al. one patient experienced no seizures, two patients had mild seizures that were not detected until 1 year of age, and three patients had seizures and focal seizures at 3 months of age. In another case, seizure onset was observed at the age of 4 years (3). The types of epilepsy also vary and include focal, epileptic spasms, tonic, myoclonic, generalized tonic-clonic, and atonic seizures in previous studies (1, 3, 13, 17). In this study, however, the transformation of seizure types, e.g., spasms to bilateral tonic-clonic seizure, was demonstrated, suggesting that the types of epilepsy in a patient with DEE91 may change, which is similar to patients with WS (2). In addition, the developmental regression observed in our case indicates that regressed development may be relatively common with DEE91. The developmental regression and loss of neck erection in our study could not be identified unless the clinical data before the first 4 months of the patient's life were available.

In our case, the variant c.1258_1259insAGTG (p. Val420Glufs^*^32) of the *PPP3CA* gene was detected in the proband, which is located on exon 12 (Figure 3B). This mutation is between the metal-binding (calmodulin binding, CaMB) and autoinhibitory (AI) domains (18, 19), resulting in the loss of the latter AI domain (Figure 3C). At present, only the following truncating variants are included in HGMD (Table 1): two non-sense variants and three frameshift variants. These truncating variants are all located between the CaMB and AI domains, which cause the main clinical features of epileptic encephalopathy, neurodevelopmental disease, and severe seizures (Table 1).

**Table 1:** Truncating mutations in the *PPP3CA* gene in the historical literature and this report.

**Variant number**	**Nucleic acid change**	**Protein change**	**Mutant type**	**Disease or phenotype**	**References**
1	1255_1256delAG	Ser419fs	Frameshift	Epileptic encephalopathy, early infantile	(13)
2	1283dupC	Thr429fs	Frameshift	Epileptic encephalopathy, early infantile	(14)
3	1290dupC	Met431fs	Frameshift	Epileptic encephalopathy	(1)
4	1324C>T	Gln442^*^	Non-Sense	Epileptic encephalopathy	(15)
5	1333C>T	Gln445^*^	Non-Sense	Neurodevelopmental disease, severe with seizures	(3)
6	1258_1259insAGTG	Val440fs	Frameshift	Seizures, generalized developmental delay, epileptic seizures, infantile spasms, feeding difficulties	This study, 2022

* means “stopping translation”.

Recently, Panneerselvam et al. revealed that all the reported truncating variants are located in a cluster within a 26-amino acid region in the regulatory domain (RD) in relatively more severe DEE91 cases. It concluded that patients with a truncating variant experienced more severe seizures with earlier onsets compared to those of patients with an LOF missense variant, while autism spectrum disorder was relatively common in the latter (20). Rydzanicz et al. described the discovery of a novel *de novo* c.1324C>T (p. (Gln442Ter) *PPP3CA* variant by WES in a boy with severe early-onset epileptic encephalopathy. Western blot experiments in patient cells (EBV-transformed lymphocytes and neural cells obtained by reprogramming) showed that the protein expression levels of both mutant and wild-type proteins were significantly reduced despite normal mRNA abundances (15). Our findings support this theory.

In conclusion, we identified a novel *PPP3CA* frameshift variant through WES, confirming the disease of the proband at the molecular level. The results expand the spectrum of pathogenic variants of DEE91. The clinical features and molecular evidence in the patient support the theory that LOF mutation in *PPP3CA* causes DEE91 and truncating variants within RD lead to severe phenotype. Based on the above results, WES has become an important method for diagnosing rare genetic diseases.

## Data availability statement

The datasets presented in this article are not readily available because of ethical and privacy restrictions. Requests to access the datasets should be directed to the corresponding author.

## Ethics statement

Written informed consent was obtained from the minor(s)' legal guardian/next of kin for the publication of any potentially identifiable images or data included in this article.

## Author contributions

JL enrolled the patient, collected, and interpreted the clinical information and wrote the manuscript. JC designed the study and corrected the manuscript. Both authors approved the final manuscript.

## Funding

This study was supported by the Department of Medical General Ward, Children's Hospital of Chongqing Medical University, Chongqing, China.

## Conflict of interest

The authors declare that the research was conducted in the absence of any commercial or financial relationships that could be construed as a potential conflict of interest.

## Publisher's note

All claims expressed in this article are solely those of the authors and do not necessarily represent those of their affiliated organizations, or those of the publisher, the editors and the reviewers. Any product that may be evaluated in this article, or claim that may be made by its manufacturer, is not guaranteed or endorsed by the publisher.
